# Circular RNA UBAP2 contributes to tumor growth and metastasis of cervical cancer via modulating miR-361-3p/SOX4 axis

**DOI:** 10.1186/s12935-020-01436-z

**Published:** 2020-07-31

**Authors:** Lingli Meng, Xiupeng Jia, Wenying Yu, Chunnian Wang, Jie Chen, Fenglei Liu

**Affiliations:** 1 Department of Histopathology, Ningbo Clinical Pathology Diagnosis Center, Ningbo, 315021 Zhejiang China; 2 Department of Experimental Pathology, Ningbo Clinical Pathology Diagnosis Center, Ningbo, 315021 Zhejiang China; 3 Department of Pathology, Gansu Provincial Hospital, No. 204, Donggang West Road, Chengguan District, Lanzhou, 730000 Gansu China

**Keywords:** circUBAP2, miR-361-3p, SOX4, Cervical cancer, Metastasis

## Abstract

**Background:**

Increasing researches have reported that circular RNA UBAP2 (circUBAP2) may be a potential prognosis biomarker and participate in the development of several cancers; however, the role of circUBAP2 in cervical cancer (CC) remains largely unclear.

**Methods:**

We applied qRT-PCR and Western blot to examine expression levels of circUBAP2, miR-361-3p, SOX4, Bax, Bcl-2, Cleaved caspase 3, N-cadherin, Vimentin and E-cadherin. Cell proliferation, apoptosis, invasion and migration were analyzed by MTT assay, Flow cytometry, and Transwell assay, respectively. The interaction between miR-361-3p and circUBAP2 or SOX4 was confirmed by luciferase reporter assay and pull-down assay. Murine xenograft model was established by injecting SiHa cells which stably transfected sh-circUBAP2.

**Results:**

CircUBAP2 was up-regulated in CC tissues and cell lines and high circUBAP2 expression predicated poor outcome. Knockdown of circUBAP2 suppressed cell proliferation, migration, invasion and EMT, while induced apoptosis in CC in vitro, and inhibited tumor growth and metastasis in vivo. MiR-361-3p directly bound to circUBAP2 or SOX4, and circUBAP2 could regulate SOX4 expression by sponging miR-361-3p in CC cells. Furthermore, rescue assay results demonstrated that the inhibitory effects of circUBAP2 knockdown on cell growth and metastasis were partially reversed by miR-361-3p down-regulation or SOX4 up-regulation in CC.

**Conclusion:**

CircUBAP2 represents a prognostic marker and contributes to tumor growth and metastasis via modulating miR-361-3p/SOX4 axis in CC, which indicates a potential therapeutic target for CC treatment.

## Highlight

1. CircUBAP2 is up-regulated in cervical cancer tissues and cell lines and highly expressed circUBAP2 predicates poor outcome.
2. CircUBAP2 silence inhibits the cervical cancer cell growth and metastasis in vitro and in vivo.
3. CircUBAP2 can regulate SOX4 expression by interacting with miR-361-3p in the cytoplasm in cervical cancer cells.
4. CircUBAP2 deletion inhibits the cervical cancer cell progression in vitro via severing as a ceRNA of miR-361-3p to regulate SOX4.

## Background

As the second most common female malignancy worldwide, cervical cancer (CC) has become the leading cause of cancer-related mortality among females in developing countries [1]. Despite advances in the screening techniques and current treatments, including surgery, radiotherapy and chemotherapy, the overall survival of CC patients remains unsatisfactory because of recurrence and metastasis [2, 3]. In recent, reports have suggested infection with high-risk type human papillomavirus (HPV), such as HPV 16 and 18, increases the risk of CC [4]. However, the viral infection is not enough to explain the progression of CC [5], the pathogenesis of CC remains vague.

Circular RNAs (CircRNAs) are novel, endogenous, non-coding RNAs that were recently identified and display widely prevalent expression in the eukaryotic transcriptome [6, 7]. CircRNAs have a covalently closed loop structure without a 5′ cap and a 3′ polyadenylated tail, which makes them resistant to regular mechanisms of linear RNA decay [8]. Previously, circRNAs were considered as accidental splicing byproducts with low abundance and little functional potential [9]. However, with the development of new technologies, circRNAs have been identified to be more abundantly expressed compared with their linear counterparts [10], and emerging evidence has revealed circRNAs regulate gene expression in many biological processes and involve in the initiation, prognosis and development of diverse diseases, including tumors [11, 12]. In recent, circRNAs are one of the novel objects which have recently been evaluated in CC, many circRNAs, like has-circ-0018289, hsa-circRNA-101996 have been identified to contribute to the progression of CC via sponging target microRNA (miRNA) [13, 14], indicating the regulatory role of circRNAs in CC tumorigenesis. CircUBAP2 is one type of circRNA; recently, accumulating researches have reported that circUBAP2 may be a potential prognosis biomarker and participate in the development of several cancers, such as osteosarcoma, lung cancer, and triple-negative breast cancer [15–17], suggesting the potential mechanism of circUBAP2 in tumor progression. However, the role of circUBAP2 in CC is still largely unclear.

Many circRNAs have been found to contain diverse types and quantities of miRNAs binding sites, which allow them to specifically sponge to miRNAs, thereby repressing miRNAs activity and promoting the relative expression of corresponding target genes of the miRNAs [18], and based on this regulation, the circRNA-miRNA-mRNA function axis has been identified. Recently, miR-361-3p was identified to be decreased in CC patients and high expressed miR-361-3p was an independent, favorable indicator of long-term overall survival in CC [19]. SOX4, a member of the SOX (sex-determining region Y-related HMG box) transcription factor family, has been revealed to be malformed expressed and involved in the progression and drug-resistant of CC [20]. All the studies indicated the potential role of miR-361-3p and SOX4 in the tumorigenesis in CC.

In this study, we investigated the expression pattern of circUBAP2 in CC tissues and cell lines and aimed to explore the regulatory role of circUBAP2 in vitro and *vivo* as well as its underlying mechanisms.

## Materials and methods

### Patients and samples

A total of 58 pairs of CC tissues and adjacent normal tissues who were undergoing surgical resection were collected from Ningbo Clinical Pathology Diagnosis Center, and all cancer tissue specimens were diagnosed as CC by pathological examination. Immediately, all fresh specimens were kept at − 80 °C for further experiments. This study was permitted by the Ethics Committee of Ningbo Clinical Pathology Diagnosis Center, and all patients had signed the written informed consent.

### Cell culture

CC cell lines C-33A, HeLa, SiHa and normal cervical epithelial cell line End1/E6E7 were purchased from the American Type Culture Collection (ATCC, Manassas, VA, USA) and then were incubated in Dulbecco’s Modified Eagle’s Medium (DMEM; Gibco, Carlsbad, CA, USA) containing with 10% fetal bovine serum (Gibco), 2 mM l-glutamine and 1% penicillin/streptomycin (Gibco) at 37 °C with 5% CO_2_.

### Quantitative real-time polymerase chain reaction (qRT-PCR)

Total RNA from cells or pull-down samples was isolated using TRIzol (Thermo Fisher Scientific, Inc., Waltham, MA, USA) following the standard procedure. PrimeScript RT reagent Kit (Takara, Dalian, China) was used to synthesize the complementary DNA in accordance with the standard protocol. The reverse transcription was conducted at 37 °C for 15 min, followed by 85 °C for 5 s, according to the manufacturer’s protocols. Then quantitative PCR was performed by using SYBR green PremixEx Taq II (Takara). The reaction mix (25 μL final volume) consisted of 12.5 μL SYBR^®^ Premix Ex TaqTM II (2×), 1 μL of each primer, 2 μL of the cDNA preparation, and 8.5 μL dH_2_O. The thermocycling conditions were as follows: 95 °C for 5 min, followed by 50 cycles of denaturation at 95 °C for 15 s and then 60 °C for 30 s. The fold changes were normalized with U6 or GAPDH and qualified by 2^−ΔΔCt^ method. The specific primer sequences were listed as follows: circUBAP2, forward 5′-AGCCTCAGAAGCCAACTCCTTTG-3′ and reverse 5′-TCAGGTTGAGATTTGAAGTCAAGAT-3′; miR-361-3p, forward 5′-CACTCCAGCTGGGTCCCCCAGGTGTGATTC-3′, and reverse 5′-CTCAACTGGTGTCGTGGAGTCGGCAATTCAGTTGAGAAATCAGA-3′; SOX4, forward 5′-GGTCTCTAGTTCTTGCACGCTC-3′, and reverse primer 5′-CGGAATCGGCACTAAGGAG-3′; U6, forward 5′-CTCGCTTCGGCAGCACA-3′, and reverse 5′-AACGCTTCACGAATTTGCGT-3′; GAPDH, forward 5′-AAGAAGGTGGTGAAGCAGGC-3′, and reverse 5′-GTCAAAGGTGGAGGAGTGGG-3′.

### Isolation of nuclear and cytoplasmic fractions

The nuclear-cytoplasmic fractionation was conducted using the Nuclear and Cytoplasmic Extraction Reagents kit (Thermo Fisher Scientific Inc.) following the manufacturer’ s protocol. After that, total RNA from the nuclear and cytoplasmic fractions was extracted and detected as described above.

### RNase R treatment

Total RNA (2 mg) was cultured with or without 3 U/mg of RNase R (Qiagen, Valencia, CA, USA) at 37 °C for 20 min. The resulting RNA was purified with the help of an RNeasy MinElute Cleanup Kit (Qiagen).

### Cells transfection

The miR-361-3p mimic, miR-361-3p inhibitor and mimic negative control (mimic-NC) were obtained from RIBOBIO (Guangzhou, China). The short hairpin RNA (shRNA) targeting circUBAP2 (sh-circUBAP2) (shRNA#1: 5′-GCTTCTAAGCTTTCTGAAACA-3′; shRNA#2: 5′-CAGCTTCTAAGCTTTCTGAAA-3′; and shRNA#3: 5′-CCCAGCTTCTAAGCTTTCTGA-3′) and shRNA scramble control (sh-NC), pcDNA and pcDNA-SOX4 overexpression vector (pcDNA-SOX4) were synthesized by Genepharma (Shanghai, China). Cell transfection was conducted by using Lipofectamine RNAiMax (Life Technologies Corporation, Carlsbad, CA, USA).

### Cell proliferation

Cell proliferation was analyzed using MTT assay (Beyotime, Shanghai, China). Transfected cells (2 × 10^3^ cells/well) were plated into 96-well plate, followed incubation with 20 μL of MTT solution for the indicated times and then DMSO was added into each well to resolve the generated formazan. Finally, the absorbance was examined at 490 nm using a microplate reader (Bio-Rad, Hercules, CA, USA).

### Cell apoptosis

The Annexin V-FITC/PI apoptosis detection kit (BD Biosciences, San Jose, CA, USA) was used to determine the apoptosis rate of HeLa and SiHa cells after transfection following the standard protocol. Briefly, transfected cells were harvested, and washed in PBS, and followed by staining with 10 μL Annexin V-FITC and PI in the dark for 15 min. Finally, cell apoptosis was analyzed using FlowJo software within 1 h on the FACScan Flow cytometer.

### Transwell assay

The in vitro cell migration and invasion assay of HeLa and SiHa cells were performed as reported previously [21]. For migration assay, transfected HeLa and SiHa cells in serum-free medium were seeded in the transwell upper chamber, and then DMEM medium harboring 10% FBS was added into the lower chamber as chemoattractant. Subsequently, the migrated cells were fixed with 5% glutaraldehyde for 10 min and stained with 0.5% toluidine blue, and counted in six randomly selected visual fields under microscope. For invasion assay, the philosophy of measurement was similar to the above steps of cell migration, except that upper transwell chambers were pre-coated with 0.8% Matrigel (BD Bioscience).

### Western blot assay

Cells were lysed by RIPA lysis buffer (Beyotime) to isolate proteins, and then protein concentrations were analyzed using the bicinchoninic acid (BCA) protein assay kit (Takara). Extracted proteins were separated by an SDS-PAGE minigel, and then transferred onto PVDF membranes. After blocking by 5% fat-free milk powder in TBST buffer, membranes were interacted with primary antibodies against SOX4 (ab80261, Abcam, Cambridge, MA, USA), PCNA (ab29, Abcam), Bax (ab32503, Abcam), Cleaved caspase 3 (ab2302, Abcam), Bcl-2 (ab692, Abcam), N-cadherin (ab18203, Abcam), Vimentin (ab92547, Abcam), E-cadherin (ab15148, Abcam), as well as GAPDH (ab181602, Abcam) at 4 °C overnight, followed by incubated with the HRP-conjugated secondary antibody (ab205781, Abcam). Finally, signal visualization was performed by ECL Substrates (Millipore, Billerica, MA, USA).

### Luciferase reporter assay

The full circUBAP2 sequences and SOX4 3′-UTR containing wild-type or mutant miR-361-3p binding sites were cloned into the pMIR-Report plasmid (Promega, Shanghai, China). Then HeLa and SiHa cells were plated into 48-well plates and co-transfected with 300 ng constructed plasmid vector and 30 nM miR-361-3p mimics or mimic-NC using Lipofectamine RNAiMax. Finally, the firefly luciferase activity was analyzed using a dual-luciferase reporter kit (Promega).

### Pull-down assay

CircUBAP2 or oligonucleotide was transcribed with Biotin RNA Labeling Mix and T7 RNA polymerase in vitro (Roche Diagnostics, Basel, Switzerland) and then sublimated with the help of an RNeasy Mini Kit (Qiagen) following the standard protocol. After that, the synthetic Bio-circUBAP2 probe and Bio-control probe were transfected into HeLa and SiHa cells. After 48 h later, cells were lysed, and total cell lysates were interacted with streptavidin-coated magnetic beads, followed by eluted, isolated and evaluated using qRT-PCR analysis.

### Xenograft experiments in vivo

BALB/c nude mice (aged 4–6 weeks, N = 10) were used to establish xenograft model. Briefly, 2 × 10^6^ SiHa cells transfected with sh-NC or sh-circUBAP2 were subcutaneously injected into 6 nude mice. Tumor volumes were calculated every 5 days and after 5 weeks, the mice were killed and tumor weights were analyzed. Additionally, 1 × 10^6^ sh-circUBAP2-SiHa or sh-NC-SiHa cells were intravenously injected into another 4 nude mice through tail vein, respectively. After 60 days, the lung tissues of each mouse were removed by surgery, and metastasis nodules of nude mice lung from each group were counted. The animal protocols were permitted by the Institutional Animal Care and Use Committee of Ningbo Clinical Pathology Diagnosis Center.

### Immunohistochemistry

The immunohistochemistry procedure was followed as described earlier [21, 22]. The paraffin sections of tumor tissue specimens of mice from the control and experimental group (4-μm-thick) were routinely deparaffinized and hydrated. Then methanolic H_2_O_2_ (45:5) was utilized to treat the sections to quench activities of endogenous peroxidase at room temperature for 30 min. After being blocked with goat serum for 1 h at room temperature, rabbit anti-Ki67 antibody (ab15580, Abcam) raised in rats for overnight at 4 °C, followed by incubation with goat anti-mouse IgG H&L (HRP) (1:800; Abcam) for 2 h at room temperature after washing with PBS. 3, 3′-Diaminobenzidine (Sigma-Aldrich, St. Louis, MO, USA) was utilized as a substrate. The sections were stained with DAB, washed with distilled water, and then counterstained in hematoxylin solution. A light microscope were used to visualize images.

### Statistical analysis

All statistical data were presented as the mean ± standard deviation (SD) and analyzed using the GraphPad Prism 7 (GraphPad Inc., San Diego, CA, USA) from at least three times independently experiment. Significant differences between groups were analyzed using one-way analysis of variance (ANOVA) followed by Dunnett’s test or Student’s *t* test. The correlations were analyzed using Pearson’s rank test. Survival curves were determined by a Kaplan–Meier plot followed by the log-rank test. *P *< 0.05 suggested statistically significant.

## Results

### CircUBAP2 is up-regulated in CC tissues and cell lines and highly expressed circUBAP2 predicts poor outcomes

**Fig. 1:**
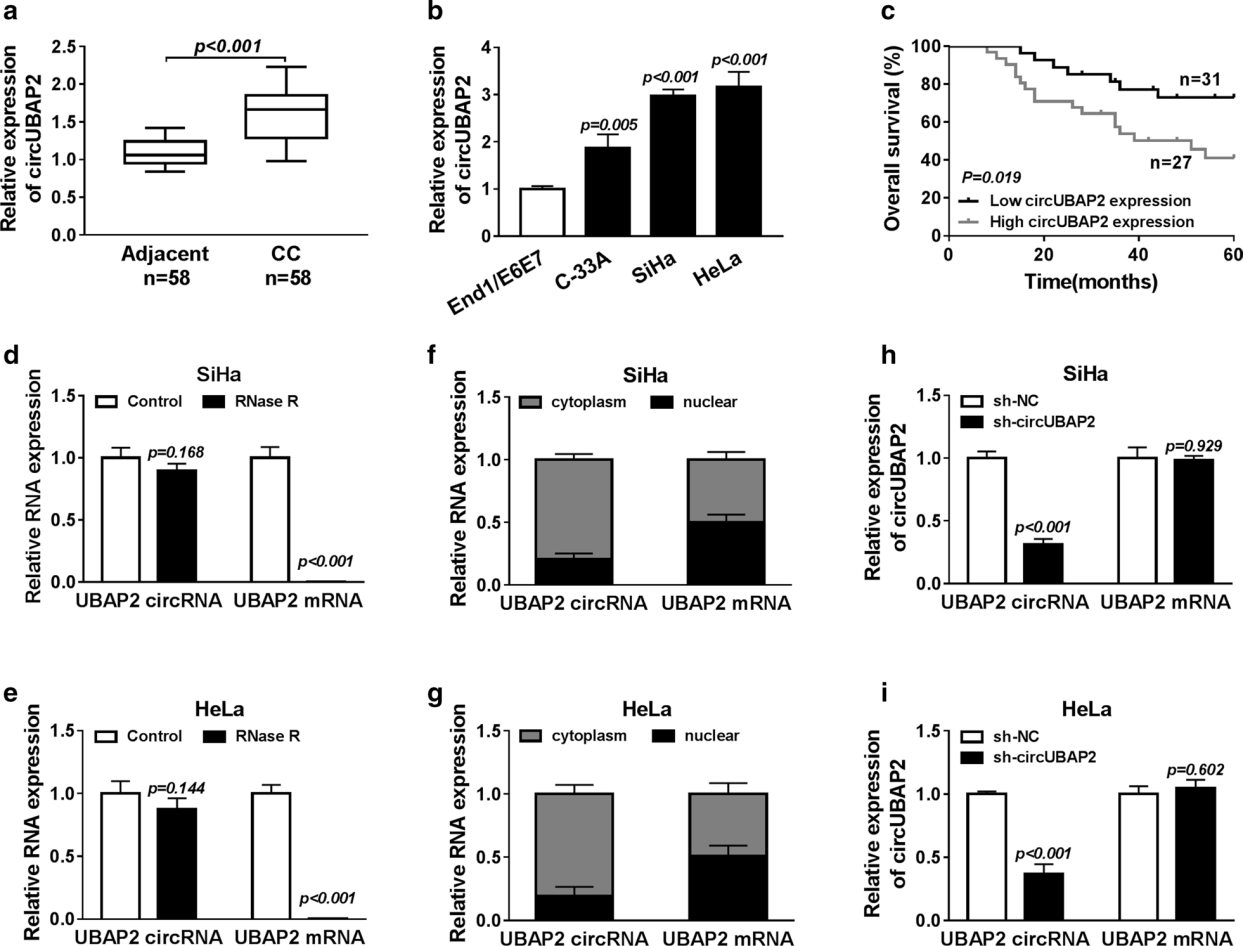
CircUBAP2 is up-regulated in CC tissues and cell lines and highly expressed CircUBAP2 predicts poor outcomes. **a**, **b** The expression of circUBAP2 was detected in CC tissues and cell lines using qRT-PCR. **c** The role of circUBAP2 in the CC prognosis was analyzed by Kaplan–Meier method. **d**, **e** qRT-PCR analysis of circUBAP2 and UBP2 mRNA after treatment with RNase R in SiHa and HeLa cells was performed. **f**, **g** CircUBAP2 and UBP2 mRNA in either the cytoplasm or the nucleus was analyzed using qRT-PCR analysis in SiHa and HeLa cells. **h**, **i** The expression of circUBAP2 and UBP2 mRNA after transfected with sh-RNAs was measured by qRT-PCR. *P < 0.05

The expression of circUBAP2 in CC tissues and cell lines was measured. Results showed that circUBAP2 was elevated in CC tissues as well as CC cell lines, including C-33A, HeLa and SiHa, compared with adjacent normal tissues and normal cervical epithelial cell line End1/E6E7 (Fig. 1a, b). Then the correlation between circUBAP2 and overall survival was analyzed and we found highly expressed circUBAP2 would result in poor survival in CC patients (Fig. 1c). All the findings suggested circUBAP2 might play an important role in CC progression. In addition, to verify the existence of circUBAP2, total RNA from proliferating SiHa and HeLa cells was treated with RNase R exonuclease and the level of RNA was detected using qRT-PCR, and then we demonstrated the circUBAP2 existed in a circular form because of the resistance to digestion by RNase R (Fig. 1d, e). Moreover, qRT-PCR analysis of nuclear and cytoplasmic RNAs indicated that circUBAP2 was preferentially localized in the cytoplasm and that UBAP2 was primarily localized in the nucleus in SiHa and HeLa cells (Fig. 1f, g). All the findings indicated that circUBAP2 was existed and levels of linear and circular UBAP2 transcript were modulated independently. Thus, to explore the potential biological functions of circUBAP2 in CC, we silenced circUBAP2 in SiHa and HeLa cells using a shRNA that lowered circUBAP2 levels without affecting the expression of the UBAP2 linear species was discovered (Fig. 1h, i).

### CircUBAP2 silence inhibits the CC cell progression in vitro

**Fig. 2:**
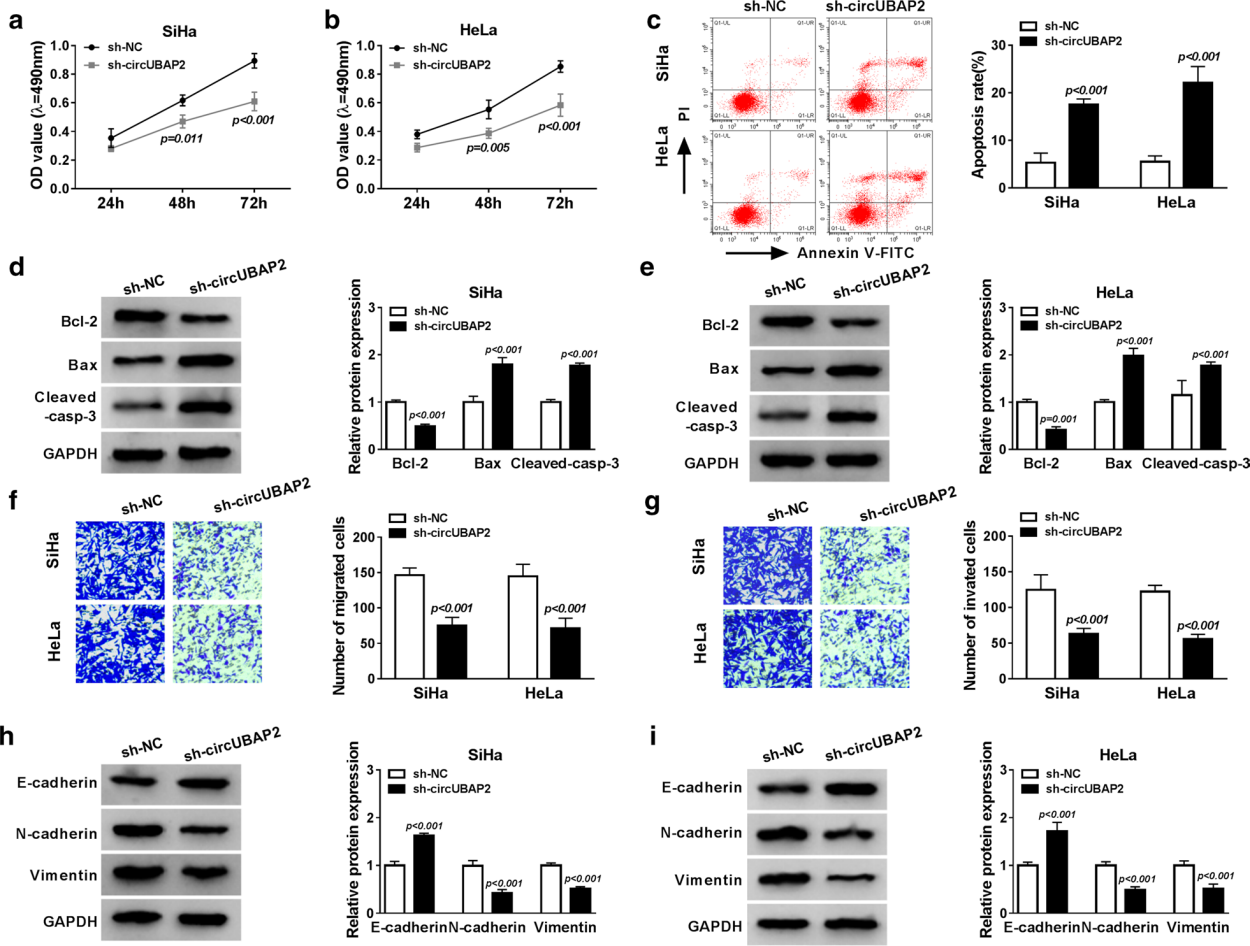
CircUBAP2 silence inhibits the CC cell progression in vitro. **a**, **b** The proliferation ability was analyzed using MTT assay in SiHa and HeLa cells. **c** The apoptosis rates were detected by Flow Cytometry in SiHa and HeLa cells. **d**, **e** The protein levels of cleaved caspase-3, Bcl-2 and Bax in SiHa and HeLa cells were examined by western blot. **f**, **g** Transwell assay was used to determine the migration and invasion abilities in SiHa and HeLa cells. **h**, **i** The levels of E-cadherin, N-cadherin and Vimentin were measured via western blot. *P < 0.05

After silencing circUBAP2 with sh-RNA, the proliferation, apoptosis, metastasis abilities of SiHa and HeLa cells were analyzed. Subsequently, MTT assay results showed knockdown of circUBAP2 inhibited cell proliferation in SiHa and HeLa cells (Fig. 2a, b), and a reduction of PCNA expression in SiHa and HeLa cells after transfection with si-circUBAP2 was also observed (Additional file 1: Fig. S1B). Then flow cytometry analysis indicated down-regulated circUBAP2 stimulated apoptosis in SiHa and HeLa cells (Fig. 2c). In the meanwhile, western blot results further validated that circUBAP2 deletion promoted apoptosis in CC cells due to the increased expression of Bax and Cleaved-casp-3 and decreased expression of Bcl-2 (Fig. 2d, e). Furthermore, a transwell assay revealed that silencing circUBAP2 suppressed cell migration and invasion in CC cells (Fig. 2f, g). Besides that, the promotion of E-cadherin level but inhibition of N-cadherin and Vimentin levels in SiHa and HeLa cells were investigated, the hallmark of epithelial-mesenchymal transition (EMT) is the loss of epithelial surface markers, most notably E-cadherin, and the acquisition of mesenchymal markers including Vimentin and N-cadherin, thus, interference of circUBAP2 also repressed EMT in CC cells (Fig. 2h, i). Therefore, we illustrated that circUBAP2 silence could suppress the CC cell progression in vitro by inhibiting proliferation, migration, invasion and EMT as well as promoting apoptosis.

### CircUBAP2 severs as a sponge of miR-361-3p and directly suppresses its expression in the cytoplasm in CC cells

**Fig. 3:**
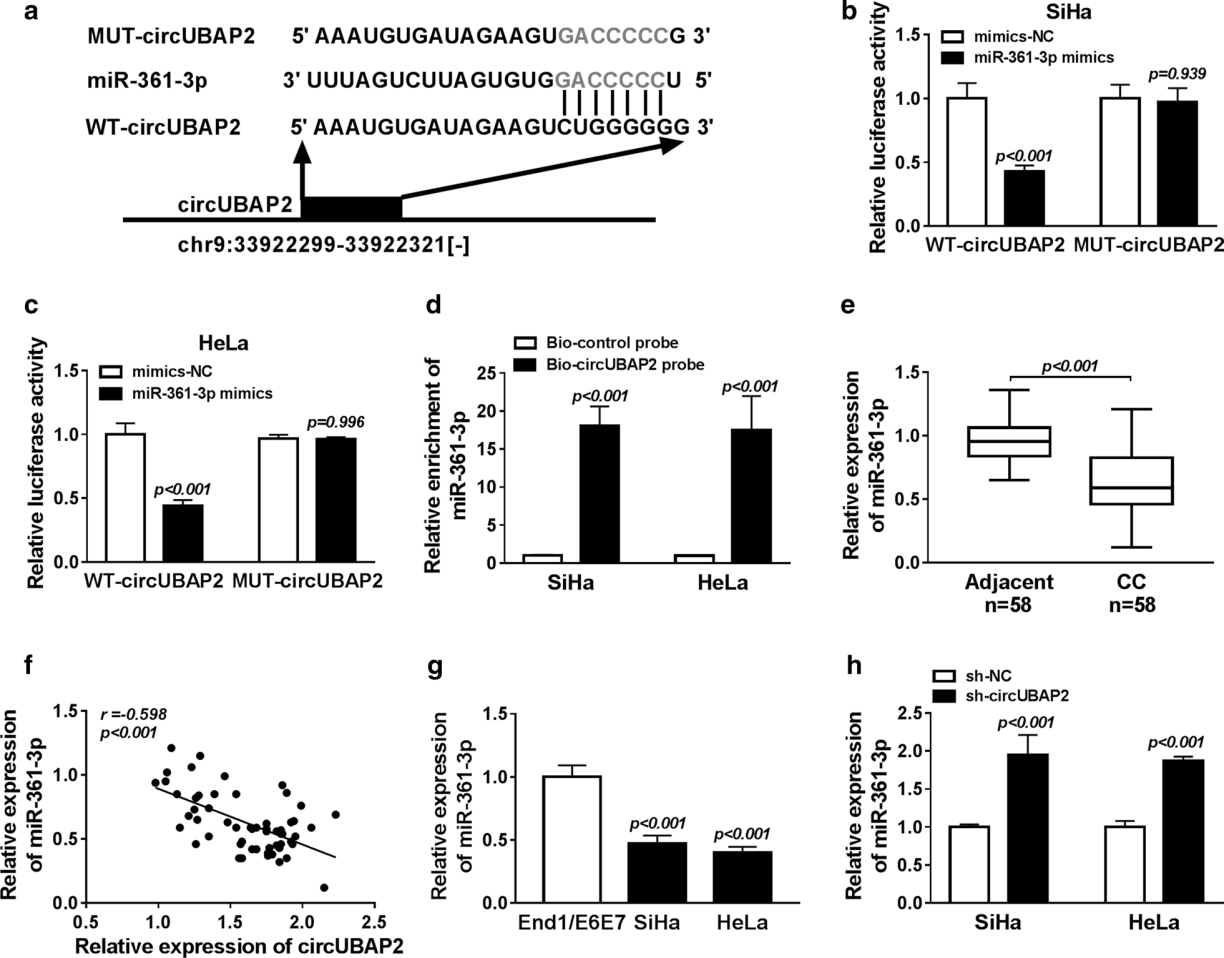
CircUBAP2 severs as a sponge of miR-361-3p and directly suppresses its expression in the cytoplasm in CC cells. **a** The predicted binding sequences between circUBAP2 and miR-361-3p was listed. **b**, **c** Luciferase reporter assay was performed in SiHa and HeLa cells co-transfected with WT-circUBAP2 or MUT-circUBAP2 and miR-361-3p mimic or mimic-NC. **d** The relative expression of miR-361-3p was evaluated by qRT-PCR after the biotinylated-circUBAP2 pull-down assays in SiHa and HeLa cells. **e**, **g** The expression of miR-361-3p was detected using qRT-PCR in CC tissues and cell lines. **f** The correlation between circUBAP2 and miR-361-3p expression was analyzed in CC patients. **h** The level of miR-361-3p was examined by qRT-PCR in SiHa and HeLa cells transfected with sh-circUBAP2. *P < 0.05

To explore the underlying molecular mechanism of circUBAP2 mediating CC cell progression, the miRNA target of circUBAP2 was predicated using the starBase2.0 program, and the putative binding sites of miR-361-3p on circUBAP2 were investigated (Fig. 3a). Then, luciferase reporter assay was performed and results showed miR-361-3p mimic transfection reduced the luciferase activities of the WT-circUBAP2 reporter vector but not MUT-circUBAP2 reporter vector in SiHa and HeLa cells (Fig. 3b, c). Moreover, The RNA pull-down assays further confirmed the direct interaction between miR-361-3p and circUBAP2 because of significant enrichment of miR-361-3p in both SiHa and HeLa cells (Fig. 3d). Subsequently, the expression of miR-361-3p was determined in CC tissues and a decrease of miR-361-3p expression was investigated in CC tissues compared with the adjacent normal tissues (Fig. 3e), besides that, a negative correlation between miR-361-3p and circUBAP2 was also discovered in CC patients (Fig. 3f). After that, down-regulated miR-361-3p expression was also detected in CC cell lines compared to the normal End1/E6E7 cell line (Fig. 3g). In addition, decreased circUBAP2 promoted miR-361-3p expression in SiHa and HeLa cells also was identified (Fig. 3h). Therefore, all the data suggested circUBAP2 served as a sponge of miR-361-3p and directly suppressed its expression in the cytoplasm in CC cells.

### MiR-361-3p directly interacts with SOX4 and negatively regulates its expression in the cytoplasm in CC cells

**Fig. 4:**
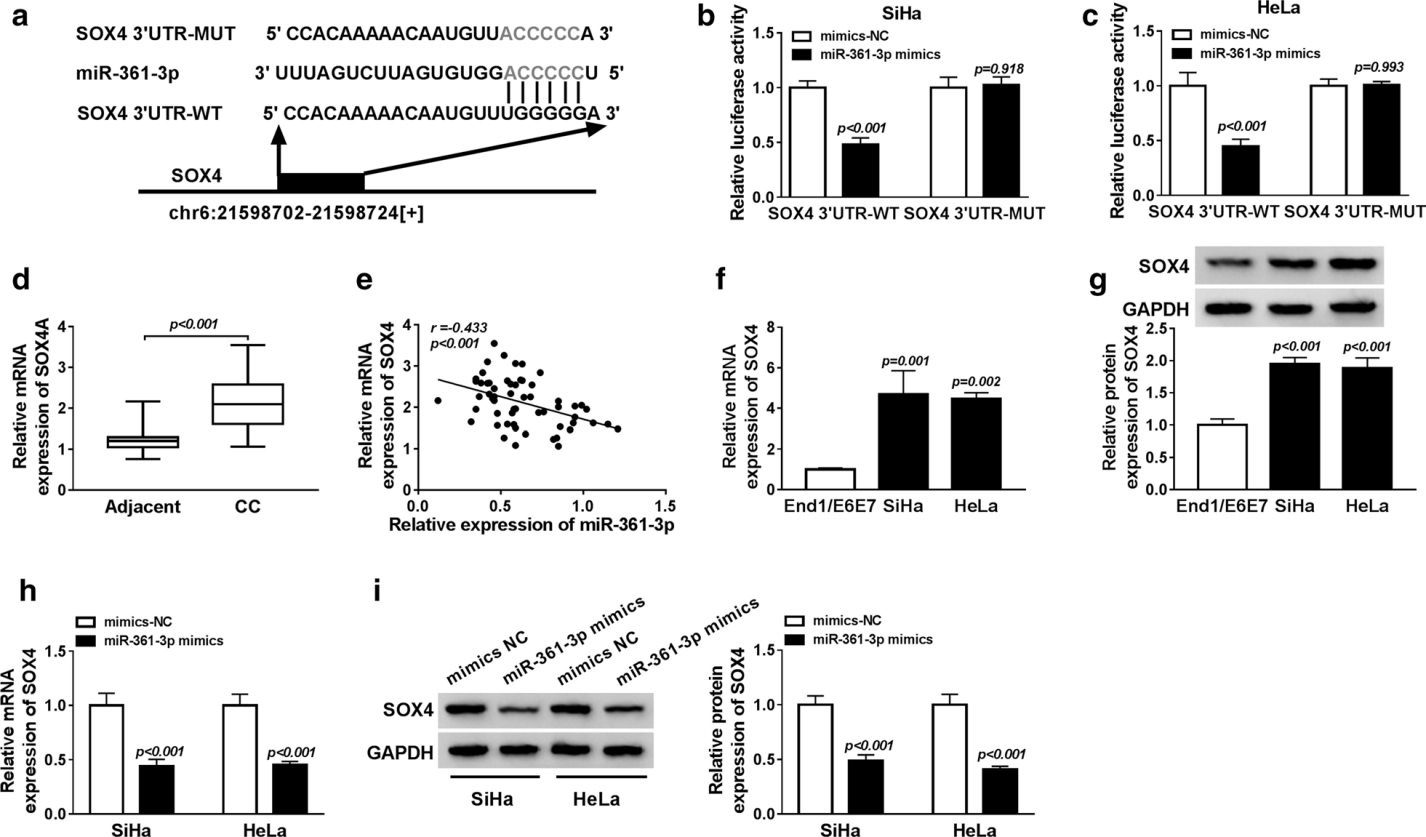
MiR-361-3p interacts with SOX4 and negatively regulates its expression in the cytoplasm in CC cells. **a** The potential binding sequence between SOX4 and miR-361-3p was listed. **b**, **c** Luciferase reporter assay was performed in SiHa and HeLa cells the co-transfected with SOX4 3′UTR-WT or SOX4 3′UTR-WT-MUT and miR-361-3p mimic or mimic-NC. **d**, **f** The mRNA expression of SOX4 was detected using qRT-PCR in CC tissues and cell lines. **e** The correlation between SOX4 and miR-361-3p expression was analyzed in CC patients. **g** Western blot was used to measure the level of SOX4 protein in SiHa, HeLa and End1/E6E7 cells. **h**, **i** The mRNA and protein expression of SOX4 was qualified using qRT-PCR or western blot in SiHa and HeLa cells transfected with miR-361-3p mimic or mimic-NC. *P < 0.05

Afterwards, we further determined the underlying mechanism of miR-361-3p and according to the prediction of the starBase2.0 program, SOX4 contained the wild or mutant type binding sequence of miR-361-3p (Fig. 4a). Subsequently, we conducted the luciferase reporter assay and the results indicated miR-361-3p mimic transfection reduced the luciferase activity of SOX4 3′UTR-WT compared with that of the control group in SiHa and HeLa cells, while the regulatory effect of miR-361-3p was disappeared in SOX4 3′UTR-MUT (Fig. 4b, c), suggesting the interaction between miR-361-3p and SOX4. Besides, qRT-PCR analysis showed a remarkably elevated expression of SOX4 mRNA in CC tissues (Fig. 4d), and, co-expression analysis showed a negative correlation between miR-361-3p and SOX4 in CC (Fig. 4e). After that, the expression of SOX4 was also measured in CC cell lines and we found, whether the mRNA or protein, SOX4 also was up-regulated in SiHa and HeLa cells compared to the End1/E6E7 cell line (Fig. 4f, g). Furthermore, miR-361-3p mimic transfection could inhibit SOX4 expression at both mRNA and protein levels in SiHa and HeLa cells (Fig. 4h, i). In all, all the data revealed that miR-361-3p interacted with SOX4 and suppressed SOX4 expression.

### CircUBAP2 deletion inhibits the CC cell progression in vitro via serving as a competing endogenous RNAs (ceRNA) of miR-361-3p to regulate SOX4

**Fig. 5:**
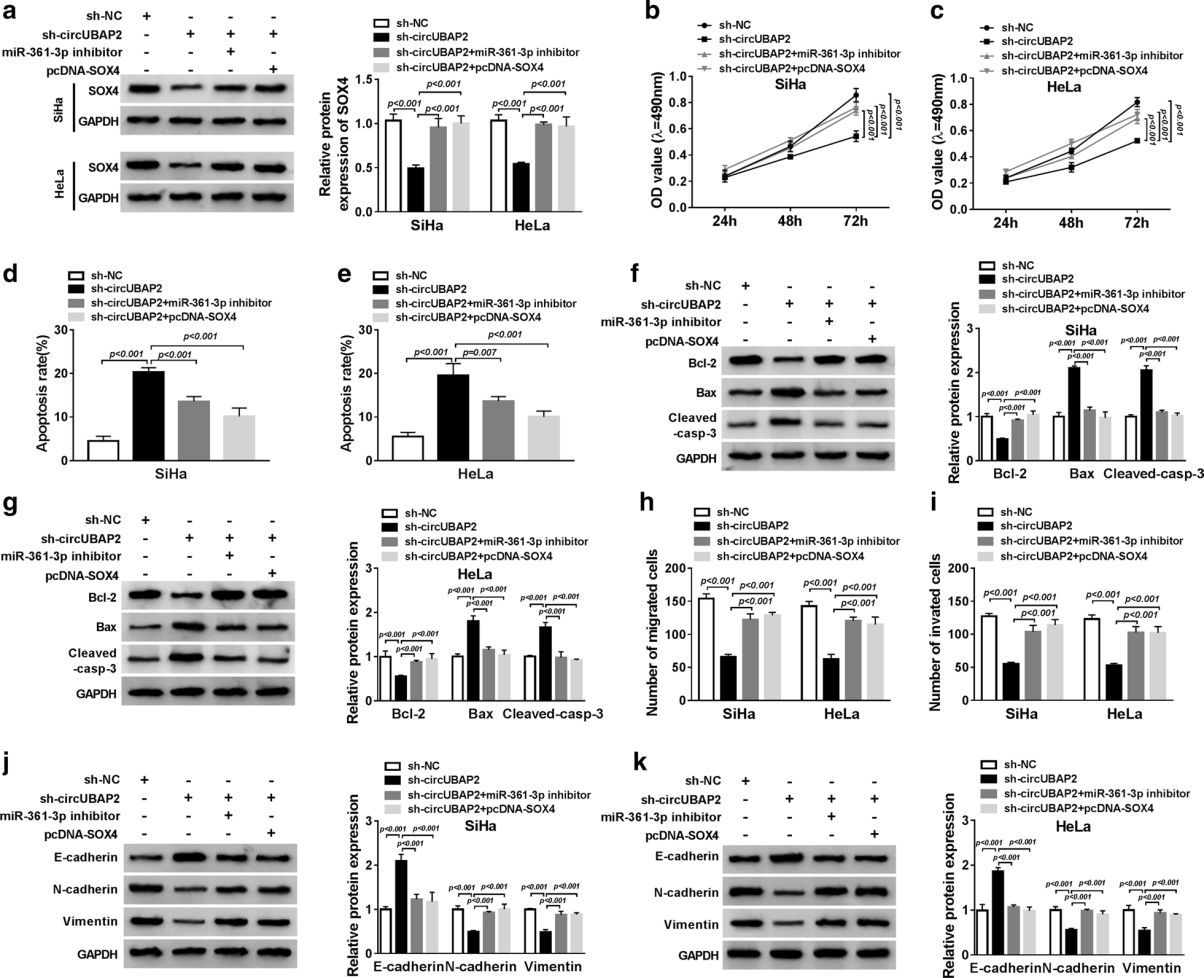
CircUBAP2 deletion inhibits the CC cell progression in vitro via severing as a ceRNA of miR-361-3p to regulate SOX4. SiHa and HeLa cells were transfected with sh-NC or sh-circUBAP2 or sh-circUBAP2 and miR-361-3p inhibitor or sh-circUBAP2 and SOX4 pcDNA. **a** The mRNA and protein expression of SOX4 in SiHa and HeLa cells was detected using qRT-PCR or western blot, respectively. **b**, **c** The proliferation ability was assessed using MTT assay in SiHa and HeLa cells. **d**, **e** The apoptosis rates were analyzed by Flow Cytometry in SiHa and HeLa cells. **f**, **g** The protein levels of cleaved caspase-3, Bcl-2 and Bax in SiHa and HeLa cells were examined by western blot. **h**, **i** The migration and invasion ability of SiHa and HeLa cells was examined by transwell assay. **j**, **k** The levels of E-cadherin, N-cadherin and Vimentin were determined using western blot. *P < 0.05

Based on the interaction between circUBAP2 and miR-361-3p or miR-361-3p and SOX4, we hypothesized that circUBAP2 deletion might affect the CC cell progression via severing as a ceRNA of miR-361-3p to regulate SOX4. Therefore, to verify this hypothesis, first, SiHa and HeLa cells were transfected with pcDNA or pcDNA-SOX4, and pcDNA-SOX4 significantly elevated SOX4 expression (Additional file 1: Fig. S1A). Next, SiHa and HeLa cells were transfected with sh-NC, sh-circUBAP2, sh-circUBAP2 + miR-361-3p inhibitor, or sh-circUBAP2 + SOX4 pcDNA. After transfection, the expression of SOX4 was examined, and we found sh-circUBAP2-induced reduction of SOX4 expression was rescued by miR-361-3p inhibitor or SOX4 pcDNA transfection in SiHa and HeLa cells (Fig. 5a), indicating circUBAP2 could act as a ceRNA of miR-361-3p to regulate downstream target SOX4 in CC cells. Afterwards, cell proliferation, migration, invasion, EMT and apoptosis abilities were analyzed. We found circUBAP2 knockdown inhibited the proliferation of SiHa and HeLa cells by reducing the expression of PCNA, while this effect was abolished by miR-361-3p down-regulation or SOX4 up-regulation (Fig. 5b, c, Additional file 1: Fig. S1B). The suppression of apoptosis of SiHa and HeLa cells caused by circUBAP2 knockdown, evidenced by the increase of apoptosis rates, Bax and cleaved caspase-3 levels, as well as the decrease of Bcl-2 level, was also reversed after the introduction of miR-361-3p inhibitor or SOX4 pcNDA (Fig. 5d–g). In the meanwhile, transwell assay and western blot analysis showed decreased miR-361-3p or overexpressed SOX4 could also reverse circUBAP2 silence-mediated inhibition of migration, invasion as well as EMT in SiHa and HeLa cells (Fig. 5h–k). Altogether, these results illustrated that circUBAP2 deletion could inhibit the CC cell progression in vitro via acting as a ceRNA of miR-361-3p to regulate SOX4.

### CircUBAP2 promotes CC cell growth and metastasis in vivo

**Fig. 6:**
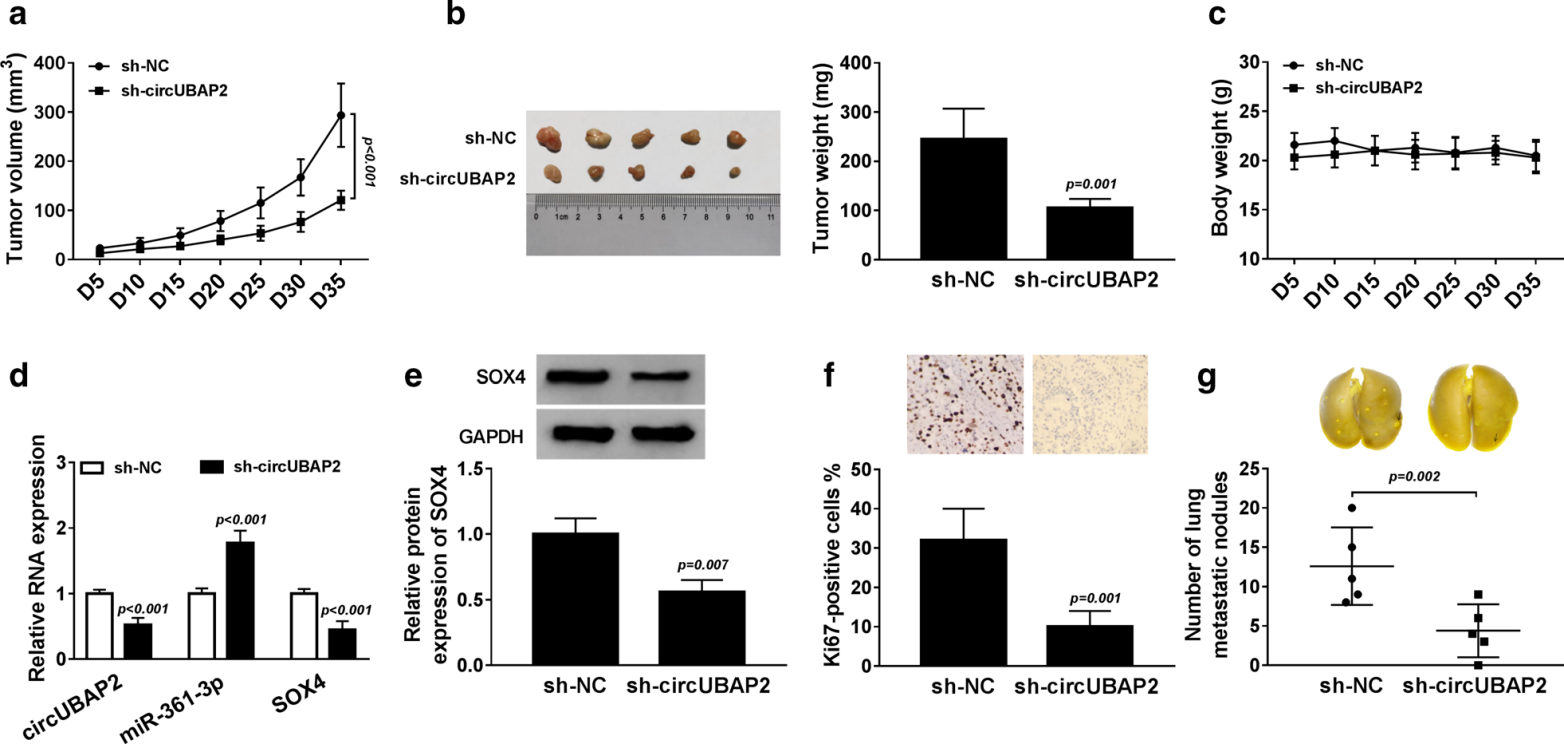
CircUBAP2 promotes CC cell growth and metastasis in vivo. **a** Tumor volume was assessed every 5 days after injection. **b** Representative photographs and average weights of dissected tumors, tumor weights were calculated in different groups at 5 weeks after injection. **c** The body weights of mice were recorded every 5 days. **d** qRT-PCR analysis of circUBAP2, miR-361-3p and SOX4 mRNA in tumor masses. **e** Western blot analysis of SOX4 protein in tumor masses. **f** Immunohistochemical analysis of Ki67 in sh-circUBAP2 and sh-NC treated tumors. **g** The metastatic nodules were counted at 8 weeks after injection. *P < 0.05

To investigate the biological functions of circUBAP2 in vivo, SiHa cells stably transfected with sh-circUBAP2, or the corresponding empty vector were subcutaneously injected into nude mice. We found that tumor lumps in the sh-circUBAP2 group were smaller than those in the sh-NC group (Fig. 6a), and after 35 days, the mice were sacrificed and the average weight of tumor lumps incubated with sh-circUBAP2-SiHa cells was significantly lighter than that of the sh-NC-SiHa cells (Fig. 6b), indicating circUBAP2 deletion significantly reduced tumor growth. Besides, we recorded the weight of mice every 5 days, and there was no significant change in body weight, suggesting shRNA has no apparent toxicity on mice (Fig. 6c). Additionally, molecular analysis suggested that sh-circUBAP2 notably reduced the expression of circUBAP2 and SOX4, but elevated miR-361-3p expression in tumor masses compared with the sh-NC group (Fig. 6d, e), revealing circUBAP2 knockdown suppressed CC tumor growth in vivo by regulating miR-361-3p and SOX4 expression. Importantly, as depicted in microphotographs and histograms in Fig. 5f, circUBAP2 knockdown suppressed the level of Ki67 in the tumor masses, further strengthening the inhibitory effect of circUBAP2 on SiHa tumor growth. In addition, to explore the effect of the metastatic inhibiting ability of circUBAP2 silence on SiHa cells, another two groups of 5 mice were intravenously injected in the tail vein with sh-circUBAP2 or sh-NC SiHa cells, respectively. After 8 weeks, the mice were sacrificed, and we demonstrated that lungs metastatic nodules were significantly fewer in sh-circUBAP2 group than that in the sh-NC group (Fig. 6g). Therefore, these results indicated that circUBAP2 could promote CC growth and metastasis in vivo.

## Discussion

CircRNAs are naturally occurring, versatile and diverse endogenous non-coding RNAs, and have covalently linked ends relative to linear RNAs [23, 24]. In recent years, accumulating evidence has investigated that circRNAs are crucial contributors in normal cell differentiation, tissue homeostasis and disease progression [25], and are implicated in several diseases, particularly in cancer [26], which may function as a diagnostic or predictive biomarker and therapeutic targets for cancers. In fact, aberrant expression of circRNAs in diverse cancer types and tissues has been identified, which involves in the oncogenesis in many cancers by sponging target miRNA or regulating downstream pathways. For example, circRNA-ITCH is significantly decreased and antagonizes lung cancer proliferation by inhibiting the activation of Wnt/β-Catenin pathway [27]. CircRNA MTO1 directly sponges to miRNA-9 to inhibit the progression of hepatocellular carcinoma [28]. CircPVT1 is highly expressed in gastric cancer tissues and may induce cell proliferation by targetedly interacting with the members of the miR-125 family [29]. However, up to now, there was no report to reveal the regulatory role of circUBAP2 in CC. Thus, in this project, we focused on the function of circUBAP2 as well as its underlying mechanism in CC progression.

Using the qRT-PCR analysis, we found circUBAP2 was increased in CC tissues and cell lines, and highly expressed circUBAP2 was related to the poor prognosis, indicating circUBAP2 might function as an oncogene in CC development. Immediately, to verify this hypothesis, circUBAP2 functions in CC cell proliferation, apoptosis and metastasis were analyzed using loss-of-function assay. As expected, circUBAP2 deletion inhibited cell proliferation, metastasis reflected by the repression of migration, invasion and EMT abilities, as well as stimulated apoptosis in CC cells. Additionally, we also further analyzed the role of circUBAP2 in vivo by using a xenograft model, which was established by injecting stably transfected sh-circUBAP2 SiHa cells and the results demonstrated that circUBAP2 deletion suppressed CC cell growth and metastasis in vivo, which further suggesting circUBAP2 acted as an oncogene in CC development.

Functional circRNAs often act as miRNA sponges to regulate protein expression of the key genes at the transcriptional or post-transcriptional level that are vital for carcinogenesis [24]. In this study, according to the prediction and confirmation of the starBase2.0 program and luciferase reporter assay, we discovered circUBAP2 directly interacted with miR-361-3p and targetedly inhibited miR-361-3p expression in CC cells. It has been revealed that miR-361-3p functions as a tumor inhibitor in many cancers such as retinoblastoma and non-small cell lung cancer by severing as a target or directly interacting with target genes [30, 31]. However, the role of miR-361-3p in CC is still overlooked. In our study, rescue assay was conducted and we identified that down-regulated miR-361-3p could reverse circUBAP2 deletion-mediated suppression on CC cell progression. Thus, we illustrated miR-361-3p might also act as a tumor suppressor in CC and circUBAP2 promoted CC progression through sponging miR-361-3p.

SOX4 has been revealed to be a downstream target of miR-133a to contribute to NEAT1/miR-133a axis-mediated CC progression [32], indicating the promotional effects of SOX4 on CC development. In this study, SOX4 was confirmed to be a target of miR-361-3p and was negatively modulated by miR-361-3p in CC cells. After that, co-expression analysis demonstrated that circUBAP2 severed as a ceRNA of miR-361-3p, thereby regulating downstream target SOX4 in CC cells in vitro and in vivo. Thus, circUBAP2/miR-361-3p/SOX4 loop was firstly investigated. In the meanwhile, we also identified that overexpressed SOX4 could attenuate circUBAP2 deletion-mediated suppression on CC cells growth and metastasis by rescue experiments. Therefore, it was suggested that circUBAP2/miR-361-3p/SOX4 axis was implicated in CC progression. SOX4 is regarded as an oncogene and has been found to be overexpressed in many types of malignancies [33]. SOX4 can activate multiple developmental pathways, including PI3K, Wnt, and TGFβ signaling, thus contributing to cell growth, EMT, and apoptosis inhibition in cancers. Besides that, SOX4 has been shown to interact with a wide variety of transcription factors, like p53, β-catenin and SMAD3, to impact tumor cell survival, stemness, and metastasis [33, 34]. These may be the potential mechanisms by which circUBAP2/miR-361-3p/SOX4 axis works; however, further researches are still needed to confirm.

Although some conclusions have been drawn from our study, the shorting comings should be pointed out. First, the expression patterns of circUBAP2, miR-361-3p and SOX4 presented are based on a limited number of tissues. Second, this study only investigated the effects of circUBAP2/miR-361-3p/SOX4 pathway on CC cell proliferation, apoptosis and metastasis, whether or not the pathway involved in other cellular processes, such as cell cycle, and metabolism, to regulate cancer progression remains vague. Regarding the shortcomings of the present study, a larger cohort of the disease analysis is essential to validate these conclusions, and new studies should be conducted to explore the roles of circUBAP2/miR-361-3p/SOX4 pathway in other cellular processes, as well as how this axis controls all these pathways.

## Conclusion

**Fig. 7:**
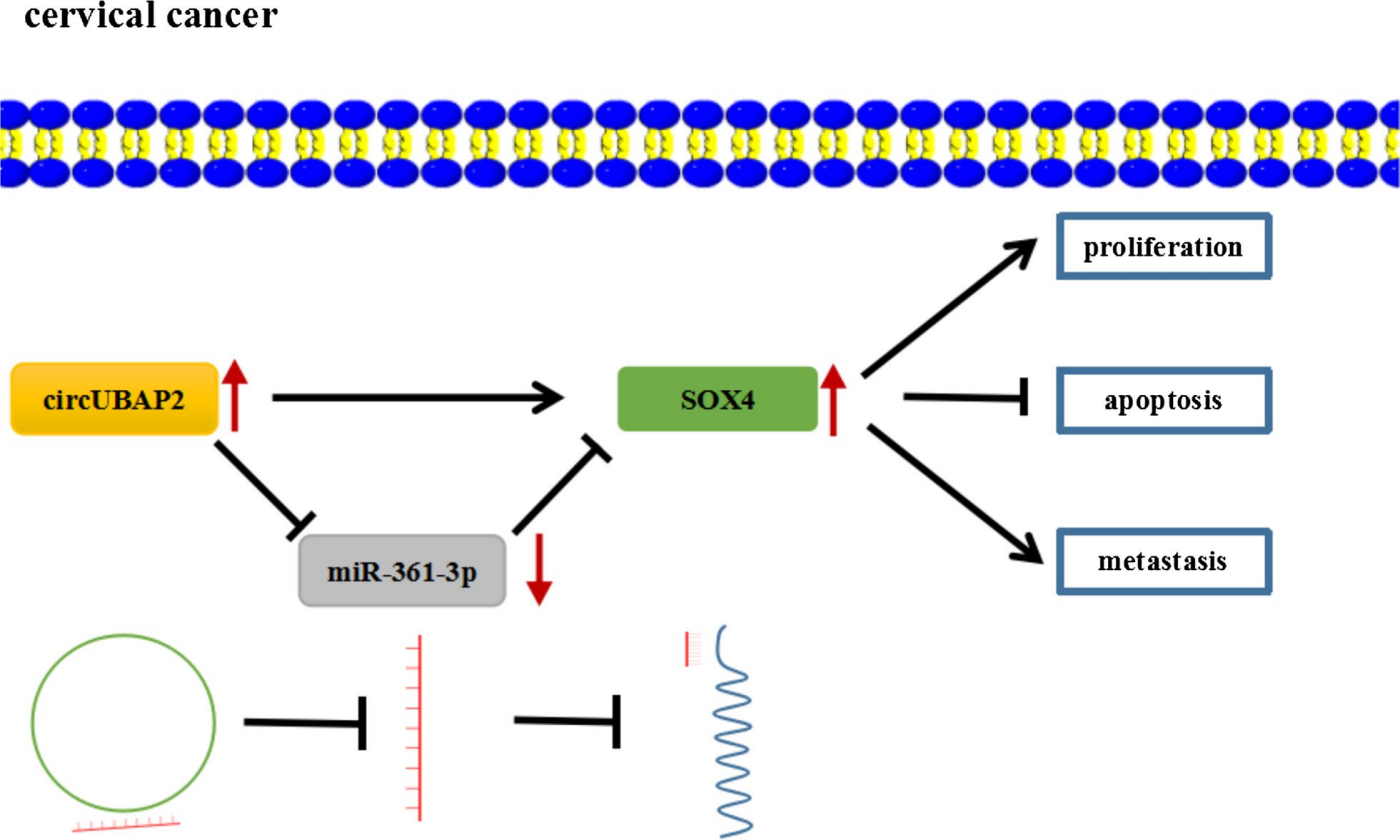
The schematic diagram of circUBAP2/miR-361-3p/SOX4 axis in CC development. CircUBAP2 up-regulated SOX4 via miR-361-3p to promote CC cell proliferation, metastasis, and inhibit cell apoptosis

To sum up, we found circUBAP2 was elevated in CC tissues and cell lines and higher expressed circUBAP2 might be a prognostic biomarker for overall survival of CC. Additionally, we firstly demonstrated circUBAP2 contributed to the CC growth and metastasis in vivo and in vitro, and then we also identified a critical circUBAP2/miR-361-3p/SOX4 axis in CC development (Fig. 7), which will help us better understand the molecular mechanism underlying the CC progression as well as prognosis and provides a novel therapeutic strategy for CC treatment.

## Supplementary information

**Additional file 1: Fig. S1** (A) The expression of SOX4 in SiHa and HeLa cells transfected with pcDNA-SOX4 or pcDNA was detected using western blot. (B) The expression of PCNA in SiHa and HeLa cells transfected with sh-NC, sh-circUBAP2, sh-circUBAP2 + miR-361-3p inhibitor, or sh-circUBAP2 + pcNDA-SOX4 was measured using western blot. *P < 0.05.

## Data Availability

All data generated or analysed during this study are included in this published article.
