# Supplementary figures and images for: Circular RNA UBAP2 contributes to tumor growth and metastasis of cervical cancer via modulating miR-361-3p/SOX4 axis
Source: Cancer Cell Int. 2020 Jul 31;20:357. doi: 10.1186/s12935-020-01436-z (PMC7393907; doi:10.1186/s12935-020-01436-z)

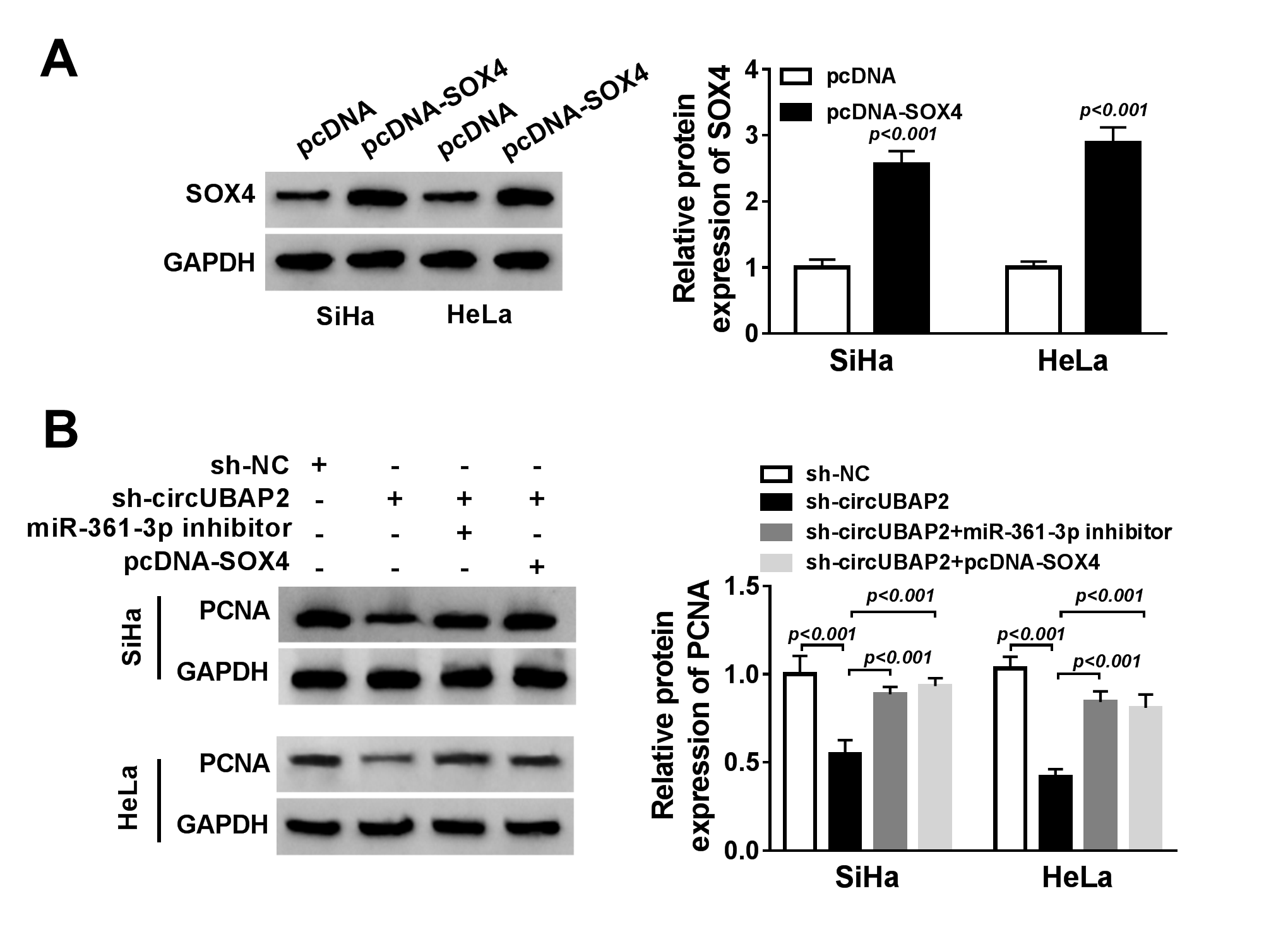

Supplement: Supplementary file 1 — Additional file 1: Fig. S1 (A) The expression of SOX4 in SiHa and HeLa cells transfected with pcDNA-SOX4 or pcDNA was detected using western blot. (B) The expression of PCNA in SiHa and HeLa cells transfected with sh-NC, sh-circUBAP2, sh-circUBAP2 + miR-361-3p inhibitor, or sh-circUBAP2 + pcNDA-SOX4 was measured using western blot. *P < 0.05. [file 12935_2020_1436_MOESM1_ESM.tif]
